# The influence of acute and chronic coronary syndrome on the gut microbiome and downstream microbiome-derived metabolites—Microbiome in acute myocardial infarction—MIAMI-Trial

**DOI:** 10.1007/s00395-025-01134-9

**Published:** 2025-08-13

**Authors:** Daniel Messiha, Erik Lange, Annika Tratnik, Astrid M.Westendorf, Miriam Rinke, Stine Lenz, Ulrike B. Hendgen-Cotta, Jan Buer, Tienush Rassaf, Christos Rammos

**Affiliations:** 1 Department of Cardiology and Vascular Medicine, West German Heart and Vascular Centre, University of Duisburg-Essen, Essen, Germany; 2 Institute of Medical Microbiology, University of Duisburg-Essen, Essen, Germany

**Keywords:** Microbiome, Coronary artery disease, Acute coronary syndrome, Chronic coronary syndrome

## Abstract

Cardiovascular diseases (CVD) are the leading cause of morbidity and mortality in the industrialized world. The gut microbiome influences CVD, through atherogenic metabolites like trimethylamine N-oxide (TMAO) or protective effects through short-chain fatty acids (SCFA) production. The specific alterations in the gut microbiome and downstream metabolites in acute coronary syndrome (ACS) and chronic coronary syndrome (CCS) remain unclear. We enrolled ACS patients within 24 h of clinical presentation with a follow-up of 28 days, using CCS patients as controls. Gut microbiome composition, downstream metabolites, and cardiovascular function were assessed at both baseline and follow-up. Microbiome-derived metabolites were analyzed and gut microbiome samples were characterized by 16S rRNA gene analysis. We enrolled 40 patients, with 20 patients each in the ACS and CCS group. Alpha diversity of the microbiome did not differ throughout the follow-up. After ACS gut microbiome composition changed during the follow-up period with increased levels of *Butyricicoccus* and *Butyricoccaceae,* a pattern not observed in the CCS cohort. Downstream analysis of microbiome-derived metabolites SCFA revealed increased serum levels of butanoic acid, while TMAO levels remained unchanged. This small prospective observational non-randomized study, suggests that ACS may trigger an enrichment of butanoic acid-producing bacteria in the gut microbiome, accompanied by an increase in serum butanoic acid levels over 28 days. No significant changes in TMAO were observed. These insights could help develop approaches to reduce the burden of CVD. As a small pilot study, these findings require validation in larger ACS cohorts. *Trial registration* NCT, NCT05456802, Registered 30 June 2022, https://clinicaltrials.gov/study/NCT05122689

## Introduction

The acute coronary syndrome (ACS) is a leading cause of morbidity and mortality in the industrialized world. It summarizes a triad of entities, including unstable angina pectoris, NSTEMI and STEMI, which typically are acute forms of manifestation of a coronary artery disease (CAD). Whereas the underlying pathophysiology is multilayered, current data show that ACS is substantially an acute inflammatory event, driven by an imbalance between pro-inflammatory and anti-inflammatory effects of the innate and adaptive immune system [27, 43]. Despite optimal medical treatment and widely implemented timely reperfusion strategies, infarct-related mortality is still high [31]. Further strategies to curb mortality are needed, with the gut microbiome having the potential to act as a key modulator here. On the other hand, chronic coronary syndrome (CCS) refers to a spectrum of clinical conditions characterized by stable, yet progressive CAD, which presents with angina triggered by exertion or stress, and reflects a chronic imbalance between myocardial oxygen supply and demand [36].

The gut microbiome is the part of the largest immunological compartment in our body. It has been introduced as a new player in the pathogenesis of atherosclerotic cardiovascular disease (ACVD), such as CAD [37, 42]. Cellular components of gut bacteria contribute to an inflammatory intestinal milieu, which in the case of gut barrier disorders, which has been described as “leaky gut”, can induce a systematic host inflammatory state [4]. Mechanistically, it has been demonstrated in preclinical models that alterations of the gut microbiome through antibiotic or probiotic treatment influenced the severity of myocardial infarction renal infarction and stroke in terms of infarct size and functional recovery [24, 30, 33, 37].

While much attention has focused on the gut microbiome as a contributor to cardiovascular pathology, recent evidence suggests the relationship may be bidirectional. The microbiome acts like a double-edged sword: Mediated by microbiome-derived metabolites, such as trimethylamine N-oxide (TMAO) or short-chain fatty acids (SCFAs), it aggravates or attenuates the progression of ACVD [13, 25, 38]. SCFAs are fatty acids with less than 6 carbons and are a result of bacterial enzymatic fermentation in the colon of indigestible nutritional elements, like dietary fiber [12]. One of the main effects of SCFAs are local and systemic anti-inflammatory effects [1]. TMAO is a proatherogenic agent that is generated by the gut microbiome from choline-rich animal products and is associated with higher risks of major adverse cardiac events (MACE) [8, 19, 21, 26].

Emerging research indicates a bidirectional relationship: the gut microbiome affects ACVD, and ACVD may, in turn, modify microbiome composition and metabolites. [39, 40].

The extent to which an acute cardiovascular event, such as ACS, affects the composition of the gut microbiome and microbiome-derived metabolites beyond the acute setting over a period of up to 28 days has not been investigated before. In the present study, we analyzed these changes to further elucidate the sequelae of ACS-induced metabolic changes. The main aim of our study is to investigate the effect of ACS on the gut microbiome composition and key microbiome-derived metabolites over a 28-day period, in comparison to patients with CCS. The primary endpoints analyzed were the changes in gut microbiome composition (including diversity indices and specific taxa abundances) and changes in circulating levels of microbiome-related metabolites (especially butanoic-acid and TMAO) from baseline to follow-up. Secondary outcomes included inflammatory biomarker levels (e.g. IL-6, CRP, leukocyte count) and basic cardiovascular parameters.

## Methods

The influence of an acute cardiovascular event such as ACS on the gut microbiome composition and microbiome-derived metabolites was investigated in a clinical trial. Patients with CCS served as controls.

Patients presenting with ACS or CCS were included into the study according to the inclusion criteria (age > 18 years, ACS or CCS, angiographically confirmed coronary artery disease with indication for PCI and written consent) and exclusion criteria (pregnancy, lactation period, antibiotic treatment within the last 3 months, chronic inflammatory bowel disease, short bowel syndrome, history of surgical treatment of the gastrointestinal system, including artificial colostomy, persistent diarrhea or vomiting within the last 3 months, current SARS-CoV2 infection, current chemotherapy or radiotherapy, simultaneous participation in another interfering study). Patients with ACS presented through the chest pain unit. Patients with CCS either presented through the chest pain unit or the outpatient clinic.

**Fig. 1:**
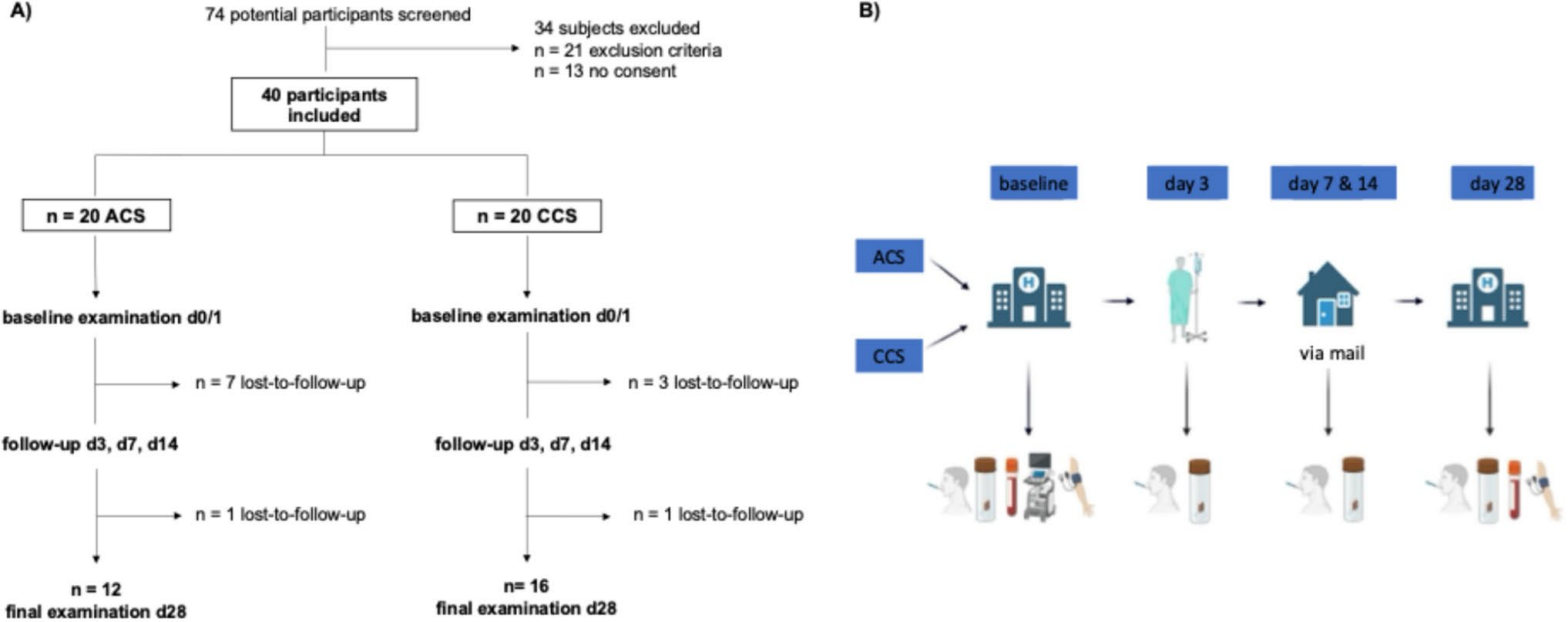
**A** Study design and **B** sample acquisition work-flow

Baseline sampling and examinations were performed within 24 h of admission to the chest pain unit and after 28 days at follow-up (Fig. 1).

The trial was conducted in accordance with the Declaration of Helsinki, was approved by the local ethics committee of the medical faculty of the University of Duisburg-Essen (21-9913-BO) and registered at Clinical Trials with the registration No: NCT05456802. Written consent of all participants was obtained prior to study enrollment.

A total of 40 patients, with 20 patients in each cohort were included. No power analysis was performed, as this study was intended as an exploratory pilot to generate hypotheses.

In the ACS cohort, 8 patients and in the CCS cohort 4 patients were lost to follow-up. Those participants who did not provide a stool or blood sample were excluded from paired comparison for the affected analysis at the affected time-points.

Examination at baseline and follow-up included measurement of basic cardiovascular parameters, including echocardiography, collection of stool samples and blood work, including routine clinical parameters like troponin-t and NT-proBNP, as well as analysis of TMAO and SCFAs panel.

Blood samples were directly analysed by our laboratory for routine care analysis. Samples for downstream metabolite analysis were centrifuged and stored according to protocol.

SCFAs levels were measured by gas chromatography mass spectrometry (GC–MS) (MS-Omics, Denmark). TMAO serum levels were measured by gas chromatography mass spectrometry (GC–MS) (Ganzimmun, Germany).

Stool samples were collected in “DNA/RNA Shield Collection & Lysis Tubes” (ZymoResearch, USA) and DNA was isolated according to the manufacturer’s protocol (ZymoBiomics DNA Miniprep Kit, USA). Sequencing of the V3/V4 region of 16S rRNA gene was performed in collaboration with *Novogene Biotech*, Beijng. Raw sequencing data were processed with the Integrated Microbial Next Generation Sequencing platform [23], where quality filtering and denoised zero-radius operational taxonomic units generation were performed. Downstream analyses were conducted using MicrobiomeStat.

Sample processing and laboratory analyses were performed blinded to group allocation.

## Statistical analysis

Statistical analysis was conducted using GraphPad Prism and MicrobiomeStat. Alpha diversity (richness, Shannon index) and metabolite levels were compared using paired t-tests or Kruskal–Wallis tests, followed by Dunn’s correction, respectively. Longitudinal taxonomic changes were assessed with LinDA, a linear model tailored for compositional microbiome data. Significance was set at *p* < 0.05. Patients without paired samples were excluded from respective comparisons.

## Results

The aim of this study was to investigate the effect of ACS on the gut microbiome composition and downstream microbiome-derived metabolites TMAO and SCFA, in comparison to a chronic cardiovascular event such as CCS. We included a total of 40 patients, with 20 patients presenting with an ACS and 20 patients presenting with a CCS. 80% of patients in the ACS group and 60% of the patients in the CCS group were male. On average, patients were 66.3 years old in the ACS group and 64.1 years old in the CCS group. The MEDAS (Mediterranean Diet Adherence Screener) score did not differ significantly between both cohorts (Table 1). Medications in both cohorts differed at baseline and follow-up in terms of antiplatelet and lipid-lowering drugs (Table 2).

**Table 1:**
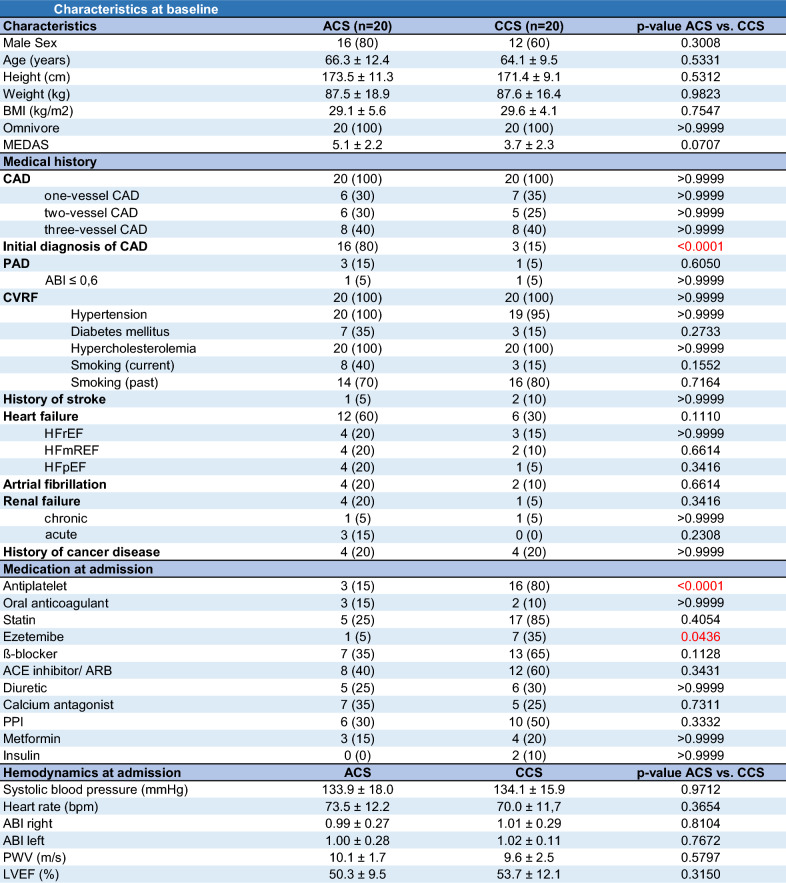
Baseline characteristics of study cohort

*BMI* body mass index, *MEDAS* Mediterranean Diet Adherence Screener, *CAD* coronary artery disease, *PAD* peripheral artery disease, *CVRF* cardiovascular risk factors, *HFrEF* heart failure with reduced ejection fraction, *HFmREF* heart failure with mid-range ejection fraction, *HFpEF* heart failure with preserved ejection fraction, *PPI* proton pump inhibitor, *ABI* ankle brachial index, *PWV* pulse wave velocity, *LVEF* left ventricular ejection fraction

**Table 2:**
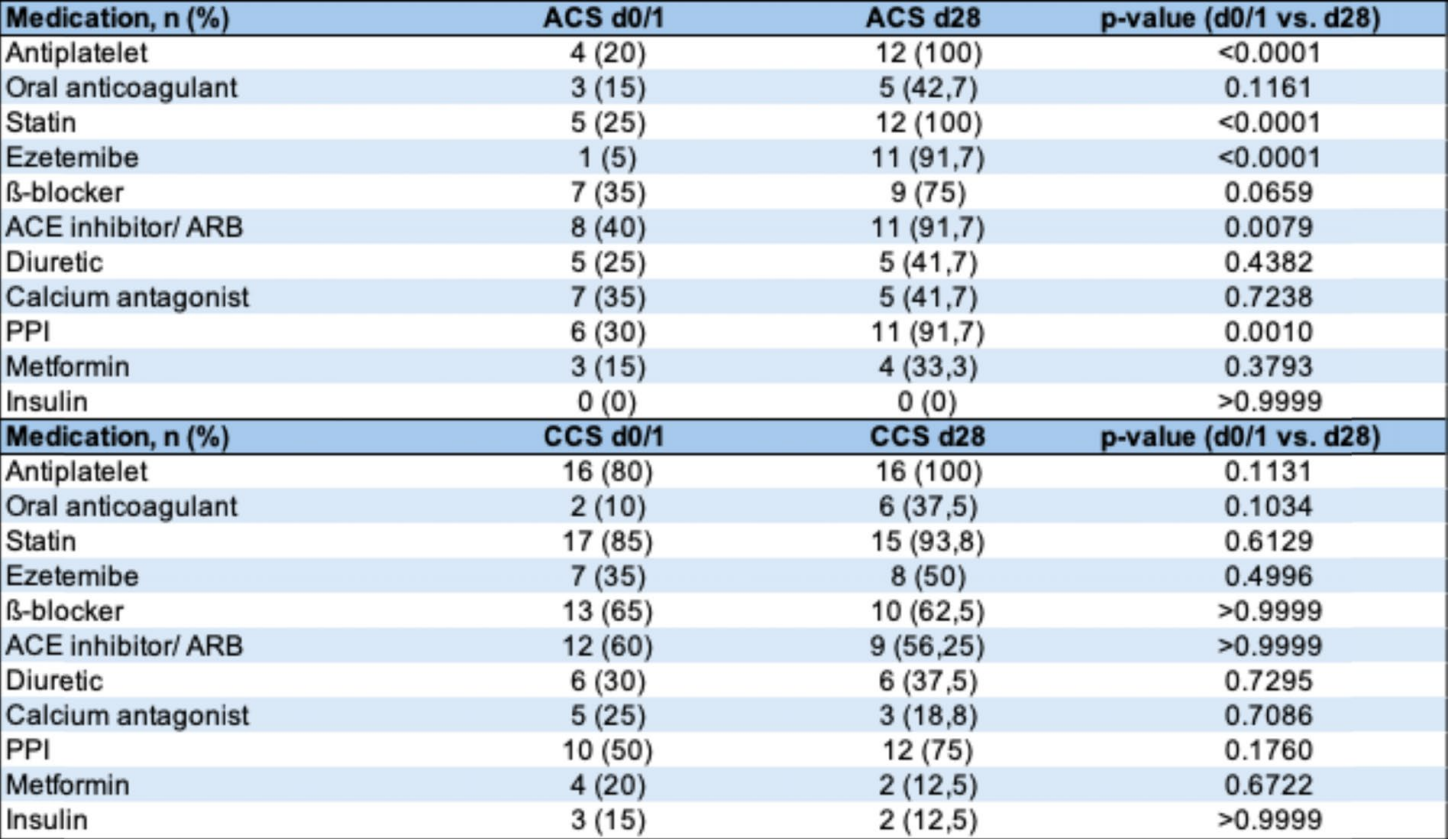
Medication at admission and at follow-up in ACS and CCS

### Inflammatory markers were significantly elevated at the onset of ACS compared to CCS patients

**Fig. 2:**
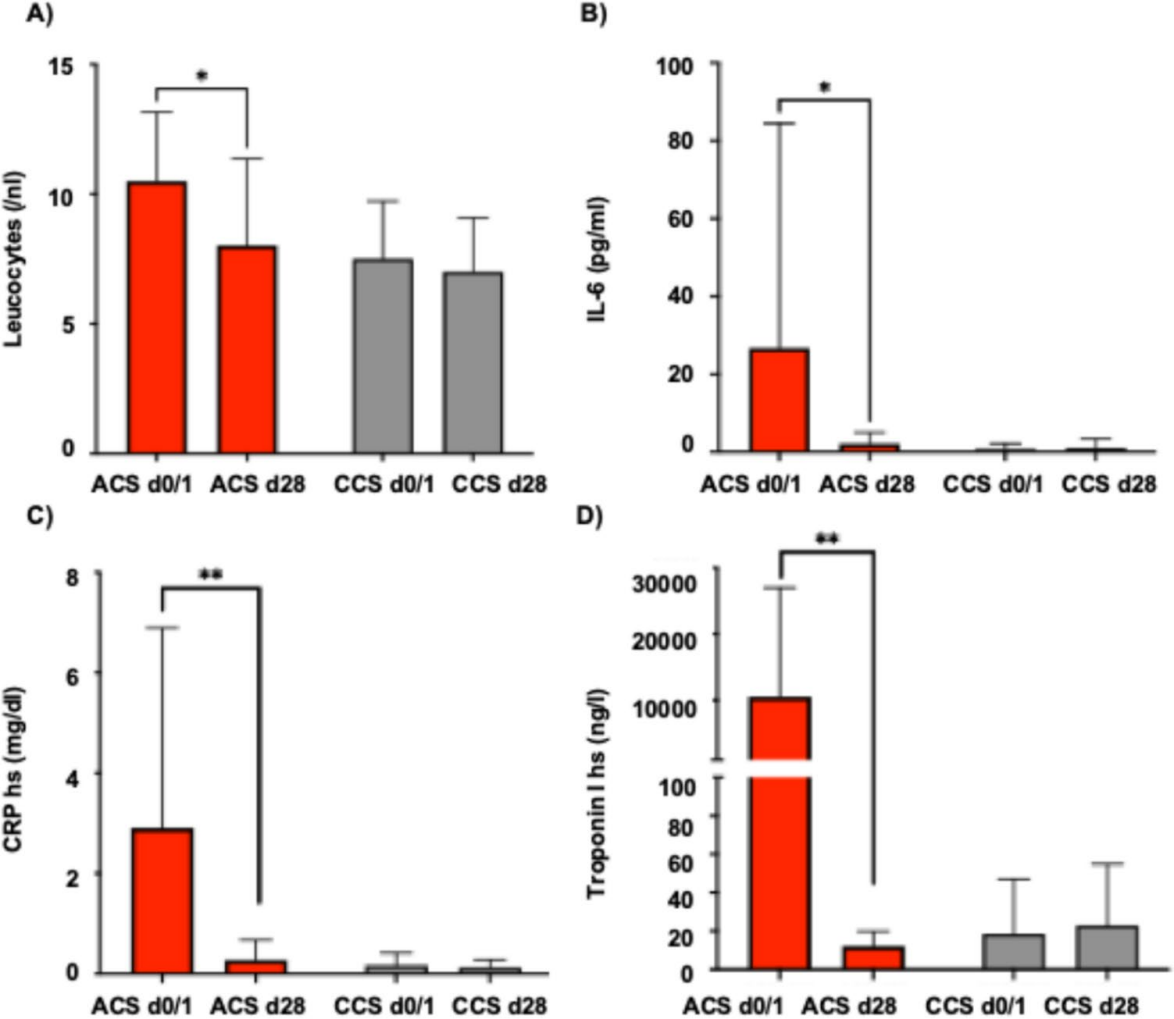
Inflammatory panel (**A-C**) including **A** leucocytes, **B** interleukin-6 and **C** high-sensitive CRP and **D** hs-Troponin I levels in patients with acute coronary syndrome (ACS) compared to patients with chronic coronary syndrome (CCS) at baseline and follow-up at 28 days. Statistical significance was calculated using the student’s t-test (* denotes *p* < 0,05, ** denotes *p* < 0.01)

Our study demonstrates that in the ACS cohort the inflammatory panel with leucocytes, interleukin-6 and high-sensitive CRP was increased upon onset of ACS and decreased over the observed period until follow-up to levels that were being observed in the CCS cohort (Fig. 2).

### Changes in gut microbiome composition over 28 days in ACS vs CCS

**Fig. 3:**
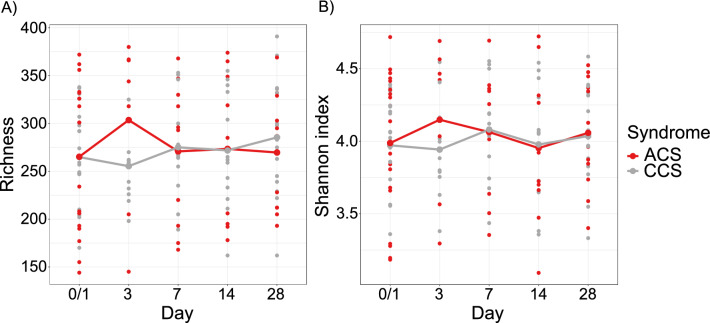
Alpha diversity metrics of patients with acute coronary syndrome (ACS) compared to patients with chronic coronary syndrome (CCS) over the observed time frame of 28 days. Bacterial (**A**) richness and (**B**) shannon index are showed with the mean value connected over time

Microbiome alpha diversity remained unchanged over the 28-day follow-up period in the ACS and CCS cohort, as demonstrated by bacterial richness and shannon index (Fig. 3).

**Fig. 4:**
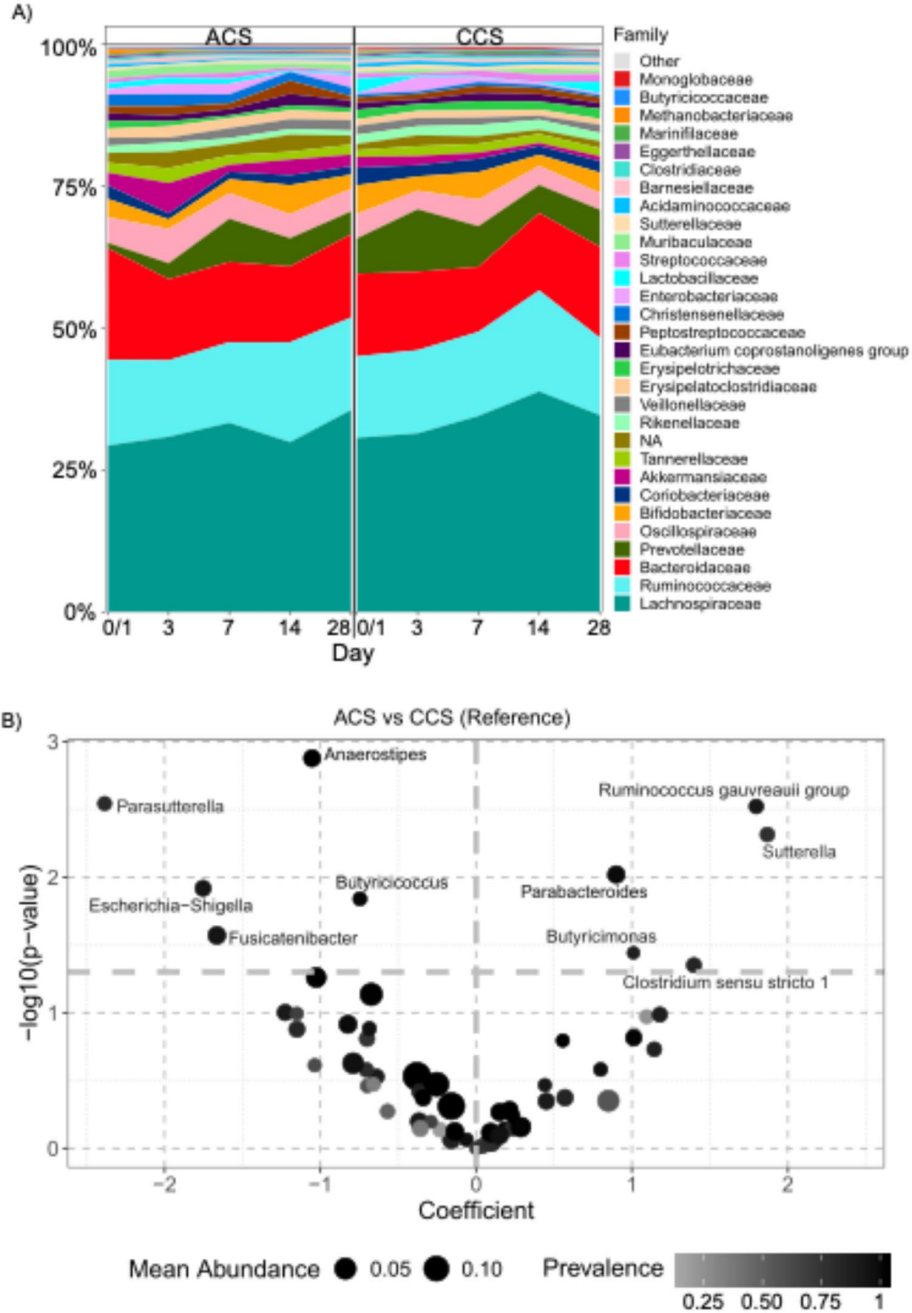
**A** Bacterial taxonomic distribution on family level of the top 30 most abundant taxa over 28 days of patients with acute coronary syndrome (ACS) compared to patients with chronic coronary syndrome (CCS). **B** Volcano plot of a trend test to analyse taxonomic changes on genus level over 28 days of patients with ACS compared to patients with CCS. Taxa mean abundance is displayed by dot size. Taxa prevalence is showed by color fade. Significant taxa are displayed above the dashed line and were calculated using the LinDA method (*p* < 0.05)

To demonstrate the changes in gut microbiome composition in ACS, we determined global changes in the gut microbiome composition. Analysis of the taxonomic distribution indicated microbial alterations in ACS compared to CCS. Further, we executed a trend test on the longitudinal microbiome data to determine significantly influenced taxa abundances over time (Fig. 4).

**Fig. 5:**
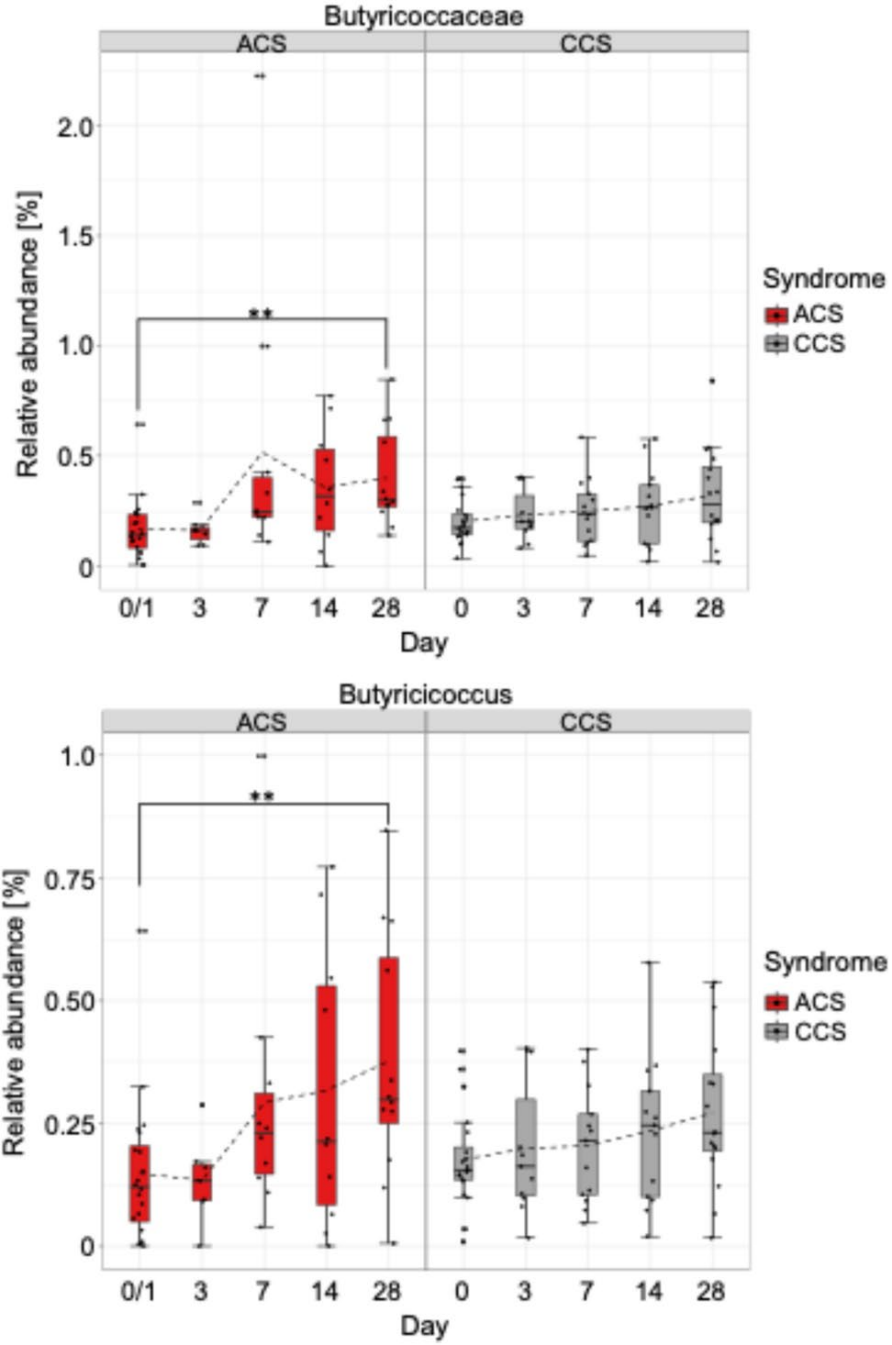
Relative abundance of individual bacterial taxa over time of patients with acute coronary syndrome (ACS) compared to patients with chronic coronary syndrome (CCS). The box spans the interquartile range (IQR, 25th-75th percentile) with the horizontal line inside representing the median. Whiskers extend to the most extreme values within 1.5 × IQR. The dashed line represents the average relative abundance trend across time points. Individual points outside the whiskers represent outliers. Significance was calculated using the Kruskal–Wallis test followed by Dunn’s multiple comparisons test (**p < 0.01)

We analysed the relative abundance of individual bacterial taxa during the follow-up period in patients with ACS compared to CCS. We detected a 2.5-fold increase in the relative abundance of *Butyricicoccus* and *Butyricicoccaceae* at the family level in the ACS cohort, whereas in the CCS cohort relative abundances of these bacterial taxa remained unchanged (Fig. 5).

### Changes in microbiome-derived metabolites over 28 days in ACS vs CCS

We then investigated SCFAs levels in the setting of ACS and CCS cohorts. Baseline SCFA levels between ACS and CCS cohort did not differ at baseline (*p* > 0.05). We found one SCFA, butanoic acid, to be differentially regulated in the ACS cohort between baseline and follow-up. SCFA levels did not change in the CCS cohort over the observed time frame.

**Fig. 6:**
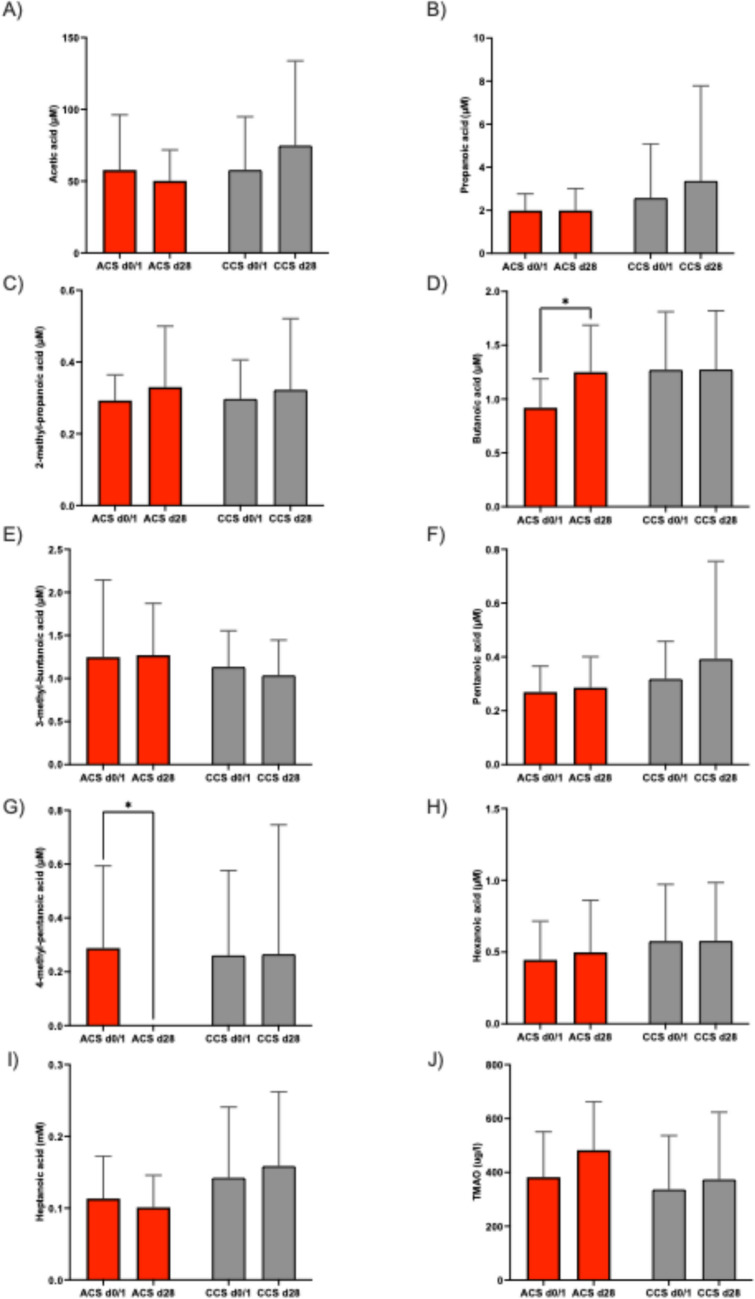
Serum levels of SCFAs (**A-I**) and TMAO (**J**) in the ACS and CCS cohort at baseline and follow-up. Serum levels of butanoic acid increased significantly during the observed time frame (*n* = 20; butanoic acid: ACS d0 vs d28 0,9 μM ± 0,3 μM vs 1,3 μM ± 0,5 μM; * depicts *p* < 0,05; ns depicts *p* > 0,05)

The microbiome-derived metabolite TMAO neither changed in the ACS nor in the CCS cohort during the observed time frame (Fig. 6).

## Discussion

Our study demonstrated that ACS as an acute atherosclerotic cardiovascular event is associated with changes in the gut microbiome composition and secondary microbiome-derived metabolites within a period of 28 days in contrast to a chronic event such as CCS.

Whereas the underlying pathophysiology of acute cardiovascular events is multilayered, current data show that ACS is substantially an acute inflammatory event. This is driven by an imbalance between pro-inflammatory and anti-inflammatory effects of the innate and adaptive immune system [10, 27, 32].

In accordance with those findings, in the ACS cohort the inflammatory panel showed an increased inflammatory activity in comparison to the CCS cohort.

Since the gut microbiome is part of the largest immunological compartment in the human body with an effect on overall cardiovascular health, we investigated the underlying mechanisms during ACS, compared to a more chronic cardiovascular setting such as CCS.

Whereas overall alpha diversity was similar between the ACS and CCS cohort, we could identify distinct changes in the composition of the gut microbiome. We detected a more than twofold increase in the relative abundance of the butanoic-acid producing bacteria *Butyricicoccus* and *Butyricoccaceae* in the ACS collective over the observed period, in contrast to the CCS collective. The butanoic-acid producing capacities of *Butyricicoccus* and *Butyricoccaceae* are well established [3, 6, 11, 14, 34, 35]. We observed a 1.4-fold increase in serum butanoic acid levels in ACS patients over the 28-day follow-up period, based on downstream metabolite analysis.

Butanoic acid is an SCFA with known gut-local and systemic anti-inflammatory effects by downregulating cytokine expression in T-cells and pro-inflammatory lamina propria macrophages [5]. Although it is the least abundant SCFA in the colon, it is the main energy source for colonocytes [7, 20]. In its function as a histone deacetylase inhibitor, butanoic acid acts as a key player in the intestinal barrier function [9]. Butanoic acid facilitates this effect by upregulation of mucin-2, which strengthens the mucosal layer, and upregulation of trefoil factors, which are important for the repair mechanism of the intestinal mucosa [18, 28]. This is in line with former studies, which demonstrated in human and mouse models that upon administration of the anti-inflammatory SCFA butanoic acid, the pro-inflammatory cytokines IFN-γ, TNF-α, IL-1β, IL-6, and IL-8 are inhibited, whereas IL-10 and TGF-β are upregulated [29]. These anti-inflammatory capacities of butanoic acid might be a pivotal mode of action during ACS to curb inflammation. Thus, the increased abundance of the butanoic-acid producing bacteria *Butyricicoccus* and *Butyricoccaceae* following ACS over a period of 28 days could be a compensatory mechanism to the overloading inflammation due to ACS. Correspondingly, inflammatory markers like leucocytes, hs-CRP and IL-6 decreased during the follow-up period in our study. In line with these findings, the cardiovascular effects of butanoic acid are well-known: It has blood-lowering effects and decreases the risk of progressive atherosclerosis disease and myocardial ischemia/reperfusion (I/R) injury [17]. In more detail, others have shown that upon oral supplementation of butanoic acid, myocardial I/R-induced inflammation, oxidative stress, and apoptosis were attenuated, presumably by influencing the gut-brain neural circuit, since those effects were diminished upon vagotomy [41].

The shift in the microbiome composition after ACS towards a higher abundance in butanoic-acid producing microbiome could pose a compensatory response that poses an interesting point for further research to reduce the burden of myocardial I/R-injury in the setting of ACS, an area where effective measurements for treatment and prevention are still limited [15, 16].

On the other side, we did not observe any significant changes in TMAO serum levels, which is in line with other findings. Others have described changes in TMAO levels beyond 4 weeks after myocardial infarction, which makes it possible we stopped just short of a period where TMAO changes would become evident [2].

This study has several limitations. Due to the study design, we can only describe correlations rather than mechanistic interactions between ACS, CCS and changes in microbiome composition. This study was intended as an exploratory pilot to generate hypotheses, and we caution that the findings need confirmation in larger cohorts with greater statistical power. Our findings have not been validated in an independent cohort thus future studies in external and larger populations are needed to verify whether these post-ACS microbiome changes are generalizable. And finally, while 16S rRNA sequencing can identify taxonomic shifts, it cannot directly measure functional capacity. We suggest that future studies use integrated meta-omics approaches to verify functional changes in microbial metabolism, based on our findings.

As medication exposure poses a potential confounder, cohorts could be matched in regard to cardiovascular medications after initial presentation. This is a limitation of this study since due to the low sample size, adding covariates to the statistical models was not feasible. Furthermore, ACS encompasses diverse pathophysiological entities—including plaque rupture, erosion, and calcified nodules [22]. Our study did not stratify patients based on these aetiologies, which may have influenced the observed gut microbiome patterns. Future studies with imaging or histopathological correlation are warranted to address this.

Taken together, we identified a specific and dynamic shift in the gut microbiome composition over 28 days in patients with ACS, distinguishing them from those presenting with CCS. Notably, this shift favoured the enrichment of butanoic-acid producing microbial populations, culminating in a 150% increase in circulating butanoic-acid levels in the ACS cohort. One hypothesis could be that its upregulation may represent an intrinsic, microbiome-mediated compensatory mechanism aimed at dampening the systemic inflammatory response characteristic of ACS. This is strongly supported by our observation of elevated inflammatory markers—including leukocytes, IL-6, and hs-CRP—at presentation.

In our study, ACS patients had lower serum butanoic-acid levels at baseline compared to CCS patients, but by day 28, ACS patient butanoic-acid levels increased significantly. We hypothesize that ACS patients may transiently lack optimal levels of anti-inflammatory microbial metabolites (such as butanoic acid) in the early phase, potentially compounding the inflammatory state post-infarct. By day 28, the recovery of butanoic acid could signify a compensatory mechanism or simply a return to equilibrium as the patient’s condition stabilizes.

This work underscores a potentially critical role of the gut microbiome in the post-ACS recovery phase in a small collective, with limited generalizability. By linking specific microbial changes to systemic anti-inflammatory outcomes, our findings suggest a hypothesis that modulation of the gut microbiome may not only be a consequence of disease but could contribute to counteract inflammation and potentially influence patient prognosis. Importantly, these insights open up new avenues for translational research, including microbiome-targeted therapies, dietary interventions, or probiotic strategies aimed at enhancing butanoic-acid production. In this broader context, our study contributes to a growing body of evidence that positions the gut-heart axis as a key therapeutic frontier in cardiovascular medicine. Further investigation is warranted to further understand the precise molecular pathways involved and to assess whether intentional modulation of the gut microbiota can improve clinical outcomes in ACS patients.

## Data Availability

All data generated or analyzed during this study are included in this published article or available upon request from the corresponding author.
